# Brain Tumor Segmentation and Survival Prediction Using Multimodal MRI Scans With Deep Learning

**DOI:** 10.3389/fnins.2019.00810

**Published:** 2019-08-16

**Authors:** Li Sun, Songtao Zhang, Hang Chen, Lin Luo

**Affiliations:** ^1^School of Innovation and Entrepreneurship, Southern University of Science and Technology, Shenzhen, China; ^2^College of Engineering, Peking University, Beijing, China

**Keywords:** survival prediction, brain tumor segmentation, 3D CNN, multimodal MRI, deep learning

## Abstract

Gliomas are the most common primary brain malignancies. Accurate and robust tumor segmentation and prediction of patients' overall survival are important for diagnosis, treatment planning and risk factor identification. Here we present a deep learning-based framework for brain tumor segmentation and survival prediction in glioma, using multimodal MRI scans. For tumor segmentation, we use ensembles of three different 3D CNN architectures for robust performance through a majority rule. This approach can effectively reduce model bias and boost performance. For survival prediction, we extract 4,524 radiomic features from segmented tumor regions, then, a decision tree and cross validation are used to select potent features. Finally, a random forest model is trained to predict the overall survival of patients. The 2018 MICCAI Multimodal Brain Tumor Segmentation Challenge (BraTS), ranks our method at 2nd and 5th place out of 60+ participating teams for survival prediction tasks and segmentation tasks respectively, achieving a promising 61.0% accuracy on the classification of short-survivors, mid-survivors and long-survivors.

## 1. Introduction

A brain tumor is a cancerous or noncancerous mass or growth of abnormal cells in the brain. Originating in the glial cells, gliomas are the most common brain tumor (Ferlay et al., 2010). Depending on the pathological evaluation of the tumor, gliomas can be categorized into glioblastoma (GBM/HGG), and lower grade glioma (LGG). Glioblastoma is one of the most aggressive and fatal human brain tumors (Bleeker et al., 2012). Gliomas contain various heterogeneous histological sub-regions, including peritumoral edema, a necrotic core, an enhancing and a non-enhancing tumor core. Magnetic resonance imaging (MRI) is commonly used in radiology to portray the phenotype and intrinsic heterogeneity of gliomas, since multimodal MRI scans, such as T1-weighted, contrast enhanced T1-weighted (T1Gd), T2-weighted, and Fluid Attenuation Inversion Recovery (FLAIR) images, provide complementary profiles for different sub-regions of gliomas. For example, the enhancing tumor sub-region is described by areas that show hyper-intensity in a T1Gd scan when compared to a T1 scan.

Accurate and robust predictions of overall survival, using automated algorithms, for patients diagnosed with gliomas can provide valuable guidance for diagnosis, treatment planning, and outcome prediction (Liu et al., 2018). However, it is difficult to select reliable and potent prognostic features. Medical imaging (e.g., MRI, CT) can provide radiographic phenotype of tumor, and it has been exploited to extract and analyze quantitative imaging features (Gillies et al., 2016). Clinical data, including patient age and resection status, can also provide important information about patients' outcome.

Segmentation of gliomas in pre-operative MRI scans, conventionally done by expert board-certified neuroradiologists, can provide quantitative morphological characterization and measurement of glioma sub-regions. It is also a pre-requisite for survival prediction since most potent features are derived from the tumor region. This quantitative analysis has great potential for diagnosis and research, as it can be used for grade assessment of gliomas and planning of treatment strategies. But this task is challenging due to the high variance in appearance and shape, ambiguous boundaries and imaging artifacts, while automatic segmentation has the advantage of fast speed, consistency in accuracy and immunity to fatigue (Sharma and Aggarwal, 2010). Until now, the automatic segmentation of brain tumors in multimodal MRI scans is still one of the most difficult tasks in medical image analysis. In recent years, deep convolutional neural networks (CNNs) have achieved great success in the field of computer vision. Inspired by the biological structure of visual cortex (Fukushima, 1980), CNNs are artificial neural networks with multiple hidden convolutional layers between the input and output layers. They have non-linear properties and are capable of extracting higher level representative features (Gu et al., 2018). Deep learning methods with CNN have shown excellent results on a wide variety of other medical imaging tasks, including diabetic retinopathy detection (Gulshan et al., 2016), skin cancer classification (Esteva et al., 2017), and brain tumor segmentation (Çiçek et al., 2016; Isensee et al., 2017; Wang et al., 2017; Sun et al., 2018).

**Figure 1:**
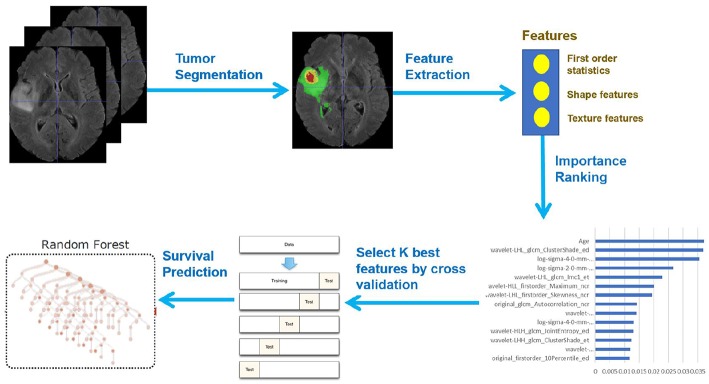
Framework overview.

In this paper, we present a novel deep learning-based framework for segmentation of a brain tumor and its subregions from multimodal MRI scans, and survival prediction based on radiomic features extracted from segmented tumor sub-regions as well as clinical features. The proposed framework for brain tumor segmentation and survival prediction using multimodal MRI scans consists of the following steps, as illustrated in Figure 1. First, tumor subregions are segmented using an ensemble model comprising three different convolutional neural network architectures for robust performance through voting (majority rule). Then radiomic features are extracted from tumor sub-regions and total tumor volume. Next, decision tree regression model with gradient boosting is used to fit the training data and rank the importance of features based on variance reduction. Cross validation is used to select the optimal number of top-ranking features to use. Finally, a random forest regression model is used to fit the training data and predict the overall survival of patients.

## 2. Materials and Methods

### 2.1. Dataset

**Figure 2:**
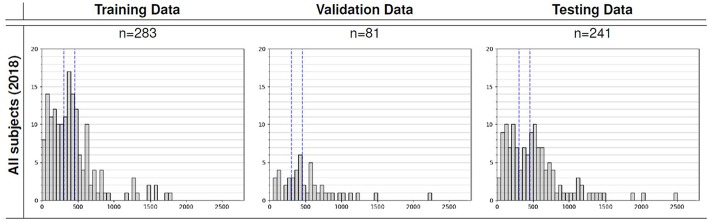
Overall survival distribution of patients across the training, validation, and testing sets.

We utilized the BraTS 2018 dataset (Menze et al., 2015; Bakas et al., 2017a,b,c, 2018) to evaluate the performance of our methods. The training set contained images from 285 patients, including 210 HGG and 75 LGG. The validation set contained MRI scans from 66 patients with brain tumors of an unknown grade. It was a predefined set constructed by BraTS challenge organizers. The test set contained images from 191 patients with a brain tumor, in which 77 patients had a resection state of Gross Total Resection (GTR) and were evaluated for survival prediction. Each patient was scanned with four sequences: T1, T1Gd, T2, and FLAIR. All the images were skull-striped and re-sampled to an isotropic 1*mm*^3^ resolution, and the four sequences of the same patient had been co-registered. The ground truth of segmentation mask was obtained by manual segmentation results given by experts. The evaluation of the model performance on the validation and testing set is performed on CBICA's Image Processing Portal ipp.cbica.upenn.edu. Segmentation annotations comprise of the following tumor subtypes: Necrotic/non-enhancing tumor (NCR), peritumoral edema (ED), and Gd-enhancing tumor (ET). Resection status and patient age are also provided. The overall survival (OS) data, defined in days, is also included in the training set. The distribution of patients' age is shown in Figure 2.

### 2.2. Data Preprocessing

Since the intensity value of MRI is dependent on the imaging protocol and scanner used, we applied intensity normalization to reduce the bias in imaging. More specifically, the intensity value of each MRI is subtracted by the mean and divided by the standard deviation of the brain region. In order to reduce overfitting, we applied random flipping and random gaussian noise to augment the training set.

### 2.3. Network Architecture

In order to perform accurate and robust brain tumor segmentation, we use an ensemble model comprising of three different convolutional neural network architectures. A variety of models have been proposed for tumor segmentation. Generally, they differ in model depth, filter number, connection way and others. Different model architectures can lead to different model performance and behavior. By training different kinds of models separately and by merging the results, the model variance can be decreased, and the overall performance can be improved (Polikar, 2006; Kamnitsas et al., 2017). We used three different CNN models and fused the result by voting (majority rule). The detailed description of each model will be discussed in the following sections.

#### 2.3.1. CA-CNN

**Figure 3:**
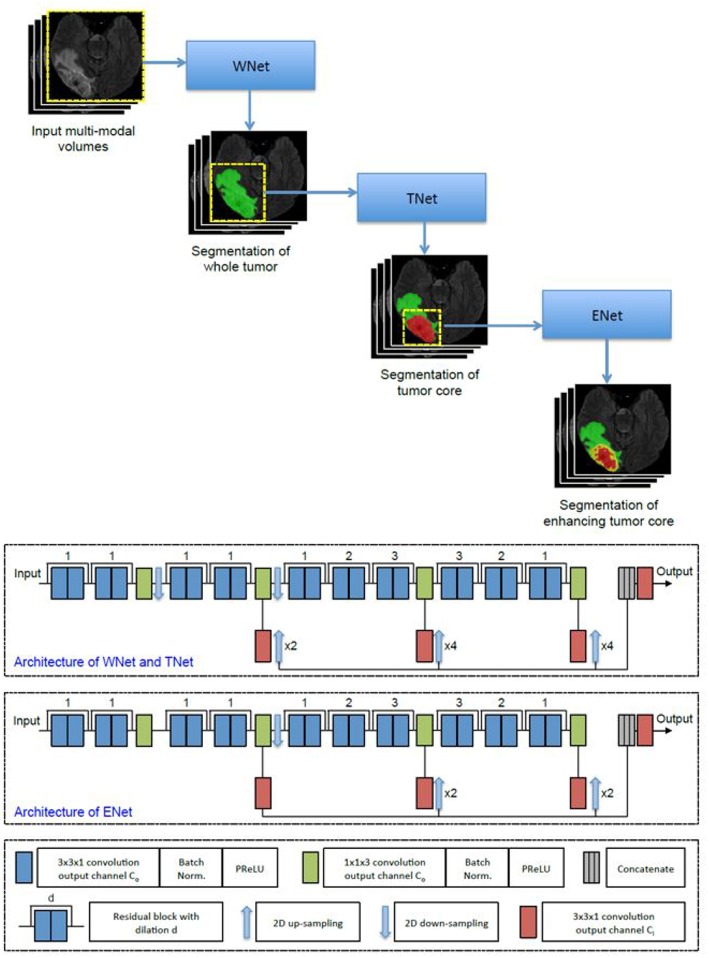
Cascaded framework and architecture of CA-CNN.

The first network we employed was Cascaded Anisotropic Convolutional Neural Network (CA-CNN) proposed by Wang et al. (2017). The cascade is used to convert multi-class segmentation problem into a sequence of three hierarchical binary segmentation problems. The network is illustrated in Figure 3.

This architecture also employs anisotropic and dilated convolution filters, which are combined with multi-view fusions to reduce false positives. It also employs residual connections (He et al., 2016), batch normalization (Ioffe and Szegedy, 2015) and multi-scale prediction to boost the performance of segmentation. For implementation, we trained the CA-CNN model using Adam optimizer (Kingma and Ba, 2014) and set Dice coefficient (Milletari et al., 2016) as the loss function. We set the initial learning rate to 1 × 10^−3^, weight decay 1 × 10^−7^, batch size 5, and maximal iteration 30*k*.

#### 2.3.2. DFKZ Net

**Figure 4:**
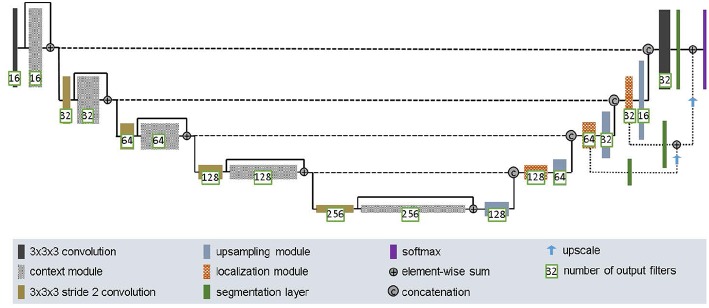
Architecture of DFKZ Net.

The second network we employed was DFKZ Net, which was proposed by Isensee et al. (2017) from the German Cancer Research Center (DFKZ). Inspired by U-Net, DFKZ Net employs a context encoding pathway that extracts increasingly abstract representations of the input, and a decoding pathway used to recombine these representations with shallower features to precisely segment the structure of interest. The context encoding pathway consists of three content modules, each has two 3 × 3 × 3 convolutional layers and a dropout layer with residual connection. The decoding pathway consists of three localization modules, each containing 3 × 3 × 3 convolutional layers followed by a 1 × 1 × 1 convolutional layer. For the decoding pathway, the output of layers of different depths are integrated by elementwise summation, thus the supervision can be injected deep in the network. The network is illustrated in Figure 4.

For implementation, we trained the network using the Adam optimizer. To address the problem of class imbalance, we utilized the multi-class Dice loss function (Isensee et al., 2017):

(1) $$\begin{array}{r} L=-\frac{2}{\left|K\right|}\underset{k\in K}{\sum }\frac{\sum_{i}u_{i\left(k\right)}v_{i\left(k\right)}}{\sum_{i}u_{i\left(k\right)}+\sum_{i}v_{i\left(k\right)}} \end{array}$$

where *u* denotes output possibility, *v* denotes one-hot encoding of ground truth, *k* denotes the class, *K* denotes the total number of classes and *i*(*k*) denotes the number of voxels for class *k* in patch. We set initial learning rate 5 × 10^−4^ and used instance normalization (Ulyanov et al., 2016a). We trained the model for 90 epochs.

#### 2.3.3. 3D U-Net

U-Net (Ronneberger et al., 2015; Çiçek et al., 2016) is a classical network for biomedical image segmentation. It consists of a contracting path to capture context and a symmetric expanding path that enables precise localization with extension. Each pathway has three convolutional layers with dropout and pooling. The contracting pathway and expanding pathway are linked by skip-connections. Each layer contains 3 × 3 × 3 convolutional kernels. The first convolutional layer has 32 filters, while deeper layers contains twice filters than previous shallower layer.

For implementation, we used Adam optimizer (Kingma and Ba, 2015), and instance normalization (Ulyanov et al., 2016b). In addition, we utilized cross entropy as the loss function. The initial learning rate was 0.001, and the model is trained for 4 epochs.

#### 2.3.4. Ensemble of Models

In order to enhance segmentation performance and to reduce model variance, we used the voting strategy (majority rule) to build an ensemble model without using a weighted scheme. During the training process, different models were trained independently. The selection of the number of iterations in the training process was based on the model's performance in the validation set. In the testing stage, each model independently predicts the class for each voxel, the final class is determined by the majority rule.

### 2.4. Feature Extraction

**Figure 5:**
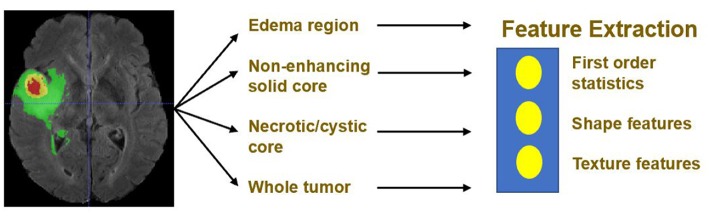
Illustration of feature extraction.

Quantitative phenotypic features from MRI scans can reveal the characteristics of brain tumors. Based on the segmentation result, we extract radiomics features from edema, non-enhancing solid core and necrotic/cystic core and the whole tumor region respectively using *Pyradiomics* toolbox (Van Griethuysen et al., 2017). Illustration of feature extraction is shown in Figure 5.

The modality used for feature extraction is dependent on the intrinsic properties of the tumor subregion. For example, edema features are extracted from FLAIR modality, since it is typically depicted by hyper-intense signal in FLAIR. Non-enhancing solid core features are extracted from T1Gd modality, since the appearance of the necrotic (NCR) and the non-enhancing (NET) tumor core is typically hypo-intense in T1Gd when compared to T1. Necrotic/cystic core tumor features are extracted from T1Gd modality, since it is described by areas that show hyper-intensity in T1Gd when compared to T1.

The features we extracted can be grouped into three categories. The first category is the first order statistics, which includes maximum intensity, minimum intensity, mean, median, 10th percentile, 90th percentile, standard deviation, variance of intensity value, energy, entropy, and others. These features characterize the gray level intensity of the tumor region.

The second category is shape features, which include volume, surface area, surface area to volume ratio, maximum 3D diameter, maximum 2D diameter for axial, coronal and sagittal plane respectively, major axis length, minor axis length and least axis length, sphericity, elongation, and other features. These features characterize the shape of the tumor region.

The third category is texture features, which include 22 gray level co-occurrence matrix (GLCM) features, 16 gray level run length matrix (GLRLM) features, 16 Gray level size zone matrix (GLSZM) features, five neighboring gray tone difference matrix (NGTDM) features and 14 gray level dependence matrix (GLDM) Features. These features characterize the texture of the tumor region.

Not only do we extract features from original images, but we also extract features from Laplacian of Gaussian (LoG) filtered images and images generated by wavelet decomposition. Because LoG filtering can enhance the edge of images, possibly enhance the boundary of the tumor, and wavelet decomposition can separate images into multiple levels of detail components (finer or coarser). More specifically, from each region, 1131 features are extracted, including 99 features extracted from the original image, and 344 features extracted from Laplacian of Gaussian filtered images, since we used four filters with sigma values 2.0, 3.0, 4.0, 5.0, respectively, and 688 features extracted from eight wavelet decomposed images (all possible combinations of applying either a High or a Low pass filter in each of the three dimensions). In total, for each patient, we extracted 1131 × 4 = 4524 radiomic features, these features are combined with clinical data (age and resection state) for survival prediction. The values of these features except for resection state are normalized by subtracting the mean and scaling it to unit variance.

### 2.5. Feature Selection

A portion of the features we extracted were redundant or irrelevant to survival prediction. In order to enhance performance and reduce overfitting, we applied feature selection to select a subset of features that have the most predictive power. Feature selection is divided into two steps: importance ranking and cross validation. We ranked the importance of features by fitting a decision tree regressor with gradient boosting using training data, then the importance of features can be determined by how effectively the feature can reduce intra-node standard deviation in leaf nodes. The second step is to select the optimal number of best features for prediction by cross validation. In the end, we selected 14 features and their importance are listed in Table 1. The detailed feature definition can be found at (https://pyradiomics.readthedocs.io/en/latest/features.html), last accessed on 30 June 2018.

**Table 1:** Selected most predicative features (WT, edema; TC, tumor core; ET, enhancing tumor; FULL, full tumor volume comprised of edema, tumor core, and enhancing tumor; N/A, not applicable).

**Extracted from**	**Name**	**Subregion**	**Score**
clinical	age	N/A	0.037375134
wavelet-LHL	glcm_ClusterShade	WT	0.036912293
log-sigma-4.0mm-3D	glcm_Correlation	TC	0.035558309
log-sigma-2.0mm-3D	gldm_LargeDependenceHighGrayLevelEmphasis	TC	0.026591038
wavelet-LHL	glcm_Informational Measure of Correlation	ET	0.022911978
wavelet-HLL	firstorder_Maximum	ET	0.020121927
wavelet-LHL	firstorder_Skewness	ET	0.019402119
original image	glcm_Autocorrelation	ET	0.014204463
wavelet-HHH	gldm_LargeDependenceLowGrayLevelEmphasis	FULL	0.014085406
log-sigma-4.0mm-3D	firstorder_Mwtian	WT	0.013031814
wavelet-HLH	glcm_JointEntropy	WT	0.013023534
wavelet-LHH	glcm_ClusterShade	TC	0.012335471
wavelet-HLL	glszm_LargeAreaHighGrayLevelEmphasis	FULL	0.011980896
original image	firstorder_10Percentile	WT	0.011803132

Unsurprisingly, age had the most predictive power among all of the features. The rest of the features selected came from both original images and derived images. We also found that most features selected came from images generated by wavelet decomposition.

### 2.6. Survival Prediction

Based on the 14 features selected, we trained a random forest regression model (Ho, 1995) for final survival prediction. The random forest regressor is a meta regressor of 100 base decision tree regressors. Each base regressor is trained on a bootstrapped sub-dataset into order to introduce randomness and diversity. Finally, the prediction from base regressors are averaged to improve prediction accuracy, robustness and suppress overfitting. Mean squared error is used as loss function when constructing individual regression model.

## 3. Results

### 3.1. Result of Tumor Segmentation

We trained the model using the 2018 MICCAI BraTS training set using the methods described above. We then applied the trained model for prediction on the validation and test set. We compared the segmentation result of the ensemble model with the individual model on the validation set. The evaluation result of our approach is shown in Table 2. For other teams' performance, please see the BraTS summarizing paper (Bakas et al., 2018). The result demonstrates that the ensemble model performs better than individual models in enhancing tumor and whole tumor, while CA-CNN performs marginally better on the tumor core.

**Table 2:** Evaluation result of ensemble model and individual models.

**Stage**	**Metric**	**Enhancing tumor**	**Whole tumor**	**Tumor core**
CA-CNN	Mean Dice	0.77682	0.90282	**0.85392**
	Mean Hausdorff95(mm)	3.3303	**5.41478**	6.56793
	Sensitivity	0.81258	0.93045	0.85305
	Specificity	0.99807	0.99336	0.99786
DFKZ Net	Mean Dice	0.76759	0.89306	0.82459
	Mean Hausdorff95(mm)	5.90781	5.60224	6.91403
	Sensitivity	0.80419	0.89128	0.81196
	Specificity	0.99833	0.99588	0.99849
3D U-Net	Mean Dice	0.78088	0.88762	0.82567
	Mean Hausdorff95(mm)	7.73567	12.63285	13.33634
	Sensitivity	0.84281	0.90188	0.81913
	Specificity	0.99743	0.99416	0.9981
Ensemble model	Mean Dice	**0.80522**	**0.90944**	0.84943
	Mean Hausdorff95(mm)	**2.77719**	6.32753	**6.37318**
	Sensitivity	0.83064	0.90688	0.83156
	Specificity	0.99815	0.99549	0.99863

*The bold values indicate the best performance*.

The predicted segmentation labels are uploaded to the CBICA's Image Processing Portal (IPP) for evaluation. BraTS Challenge uses two schemes for evaluation: Dice score and the Hausdorff distance (95th percentile). Dice score is a widely used overlap measure for pairwise comparison of segmentation mask *S* and *G*. It can be expressed in terms of set operations:

(2) $$\begin{array}{r} Dice=\frac{2|S\;\cap \;G|}{\left|S|\;+\;|G\right|} \end{array}$$

Hausdorff distance is the maximum distance of a set to the nearest point in the other set, defined as:

(3) $$\begin{array}{r} d_{H}\left(X,Y\right)=\max\left\{\underset{x\in X}{\sup}\underset{y\in Y}{\inf}d\left(x,y\right),\underset{y\in Y}{\sup}\underset{x\in X}{\inf}d\left(x,y\right)\right\} \end{array}$$

where *sup* represents the supremum and *inf* the infimum. In order to have more robust results and to avoid issues with noisy segmentation, the evaluation scheme uses the 95th percentile.

**Figure 6:**
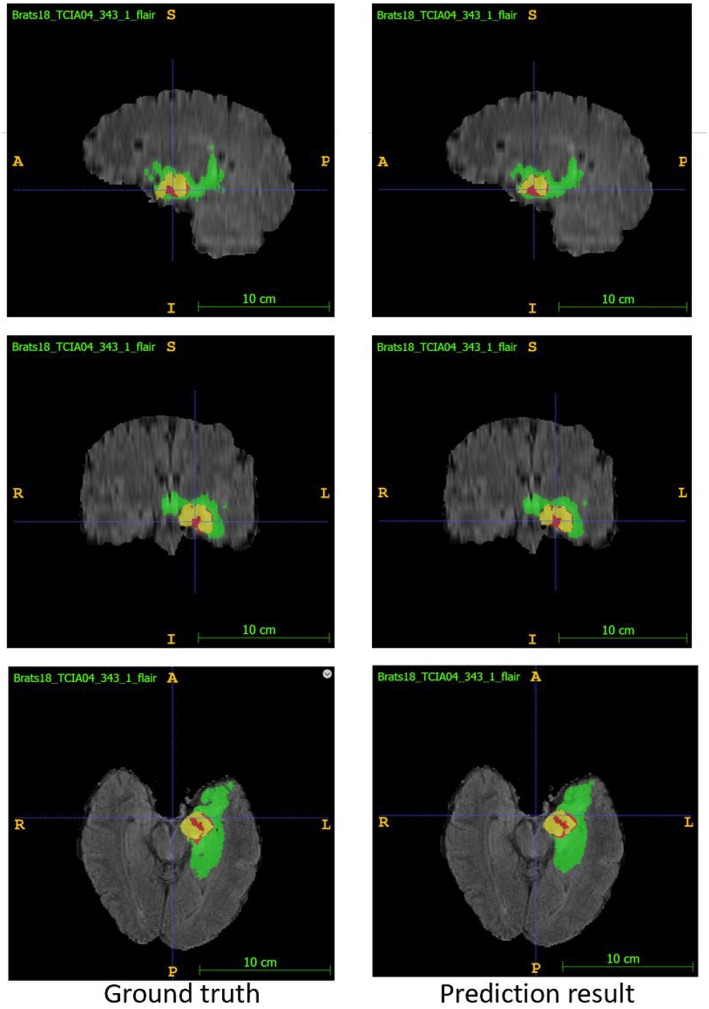
Examples of segmentation result compared with ground truth. Image ID: TCIA04_343_1, Green:edema, Yellow:non-enhancing solid core, Red:enhancing core.

In the test phase, our result ranked 5th out of 60+ teams. The evaluation result of the segmentation on the validation and test set are listed in Table 3. Examples of the segmentation result compared with ground truth are shown in Figure 6.

**Table 3:** Evaluation result of ensemble model for segmentation.

**Stage**	**Metric**	**Enhancing tumor**	**Whole tumor**	**Tumor core**
Validation	Mean Dice	0.8052	0.9044	0.8494
	Mean Hausdorff95(mm)	2.7772	6.3275	6.3732
Testing	Mean Dice	0.7171	0.8762	0.7977
	Mean Hausdorff95(mm)	4.9782	7.2009	6.4735

### 3.2. Result of Survival Prediction

Based on the segmentation result of brain tumor subregions, we extracted features from brain tumor sub-regions segmented from MRI scans and trained the survival prediction model as described above. We then used the model to predict patient's overall survival on the validation and test set. The predicted overall survival was uploaded to the IPP for evaluation. We used two schemes for evaluation: classification of subjects as long-survivors (>15 months), short-survivors (<10 months), and mid-survivors (between 10 and 15 months) and median error (in days). In the test phase, we ranked second out of 60+ teams. The evaluation results of our method are listed in Table 4. For other teams' performance, please see the BraTS summarizing paper (Bakas et al., 2018).

**Table 4:** Evaluation result of survival prediction.

**Stage**	**Classification accuracy**	**Median error**
Validation	46.4%	217.92
Test	61.0%	181.37

## 4. Discussion

In this paper, we present an automatic framework for the prediction of survival in glioma using multimodal MRI scans and clinical features. First, a deep convolutional neural network is used to segment a tumor region from MRI scans, then radiomics features are extracted and combined with clinical features to predict overall survival. For tumor segmentation, we used ensembles of three different 3D CNN architectures for robust performance through voting (majority rule). The evaluation results show that the ensemble model performs better than individual models, which indicates that the ensemble approach can effectively reduce model bias and boost performance. Although the Dice score for segmentation is promising, we noticed that the specificity of the model is much higher than the sensitivity, indicating an under-segmentation of the model. For survival prediction, we extracted shape features, first order statistics, and texture features from segmented tumor sub-region, then used a decision tree and cross validation to select features. Finally, a random forest model was trained to predict the overall survival of patients. The accuracy for three-class classification is 61.0%, which still leaves room for improvement. Part of the reason is that we only had a very limited number of samples (285 patients) to train the regression model. In addition, imaging and limited clinical features may only explain patients' survival outcome partially, too. In the future, we will explore different network architectures and training strategies to further improve our result. We will also design new features and optimize our feature selection methods for survival prediction.

## Data Availability

The datasets analyzed for this study can be found in the BraTS 2018 dataset https://www.med.upenn.edu/sbia/brats2018/data.html.

## Author Contributions

LS and SZ performed the analysis and prepared the manuscript. HC helped with the analysis. LL conceived the project, supervised and funded the study, and prepared the manuscript.

### Conflict of Interest Statement

The authors declare that the research was conducted in the absence of any commercial or financial relationships that could be construed as a potential conflict of interest.
